# Magnitude and determinants of male partner involvement in PMTCT service utilization of pregnant women attending public health facilities of Ethiopia, 2021: a systematic review and meta-analysis

**DOI:** 10.1186/s12981-022-00436-5

**Published:** 2022-02-16

**Authors:** Tamirat Melis, Yohannes Fikadu

**Affiliations:** 1 Department of Public Health, College of Medicine and Health Science, Wolkite University, Wolkite, Ethiopia; 2 Department of Midwifery, College of Medicine and Health Science, Wolkite University, Wolkite, Ethiopia

**Keywords:** Magnitude, Male partner involvement, PMTCT, HIV/AIDS

## Abstract

**Background:**

Infant antiretroviral prophylaxis has an important role in reduction of Human immune virus transmission from mother to child during the postpartum period. Male partner involvement was considered as a priority aforementioned area needs to be enhanced in Prevention of Mother-To Child Transmission (PMTCT). PMTCT service utilization can minimize the risk of the transmission of HIV from mother to child and related mortalities. Adequate utilization and adherence to this service has been challenging for some of the women if their partners are not aware or do partners do not support the women. The aim of this study is to assess the magnitude and determinants of male involvement in PMTCT service in Ethiopia.

**Methods and materials:**

We had conducted an extensive search of literature as indicated in the guideline of reporting systematic review and meta-analysis (PRISMA). We had used PubMed, Google Scholar, and cross reference for searching articles. We had used the Joanna Briggs Institute (JBI) Meta-Analysis of Statistics Assessment and Review Instrument for critical appraisal of studies. Met-analysis and meta-regression were computed to present the pooled prevalence and determinants of male partner involvement with a 95% confidence interval using Revman.

**Results:**

Among a total of 338 studies, 11 studies were included in this analysis. The estimated pooled magnitude of male partner involvement was 40% (95% CI: 29.11–50.69). Knowledge of husband on PMTCT (2.30, 95% CI 1.75, 3.02), perceived responsibility for the women (4.22, 95% CI 2.31, 7.71), being government employee (2.89, 95% CI 2.02, 4.12), cultural barriers (3.44, 95% CI 2.54, 4.65) and educational status of husband (2.4, 95% CI 1.79, 3.50) were the determinants of pooled estimates of male partner involvement in PMTCT activities.

**Conclusion:**

The pooled prevalence of male partner involvement was lower than the study conducted in sub Saharan Africa. Knowledge of husband on PMTCT, perceived responsibility for women, occupational status, cultural barriers and educational status of husband were determinants of male partner involvement. Therefore, the existing strategies to improve male involvement should be strengthened.

## Background

Transmission of HIV from mother-to-child remains a significant problem in the developing world regardless of the development and growing accessibility of effective prevention methods [1]. Male partner involvement was considered as a priority aforementioned area needs to be enhanced in Prevention of Mother-To Child Transmission (PMTCT) [2]. World Health Organization (WHO) promotes four pronged strategies to prevent the mother to child transmission of HIV. One of the strategies is involving both partners since the primary prevention throughout the care and treatment of HIV positive woman’s family [3].

Even though, the Joint United Nations Programme on HIV/AIDS(UNAIDS) report of 2016 has shown a propitious reduction in the rate of new HIV infections in children, the number of children infected with HIV still remains unacceptably high, which is around 150,000 cases per year [4]. If mothers had good adherence in providing antiretroviral prophylaxis which is prescribed by health professionals for their infants, the risk of vertical transmission of HIV reduces to less than 5% [5]. However, in condition of complex sociocultural differences especially in SSA, mothers adherence to antiretroviral prophylaxis and the uptake of other PMTCT services are strongly influenced by the involvement of their male partners [6].

In addition, only about 35–50% of the pregnant women, in Africa including Ethiopia, are enforced to attend ANC by their partner [7, 8]. The magnitude of male partner involvement in attending at least one antenatal care has ranged from 32% to 64.5% in sub-Saharan Africa [9, 10] while which was 6–58.3% in Ethiopia [11, 12].

Male partners involvement in PMTCT can reduce the risk of transmission of HIV from pregnant women to their babies and it can make progress in uptake of activities to prevent the vertical transmission of HIV [6, 13, 14]. In addition, it can improve PMTCT services utilization [15]. Male partner involvement in perinatal care has significant role in improving all perinatal care services utilization, breastfeeding status and new born care [16, 17]. Wife’s Awareness on ANC appointment, having discussion with wife about HCT in her past pregnancy, ever receiving HCT together with the partner, providing financial support to partner to attend health facilities to receive ANC, condom utilization during pregnancy if recommended by health professionals were the role of partners in PMTCT service utilization [2]. Socio-demographic characteristics such as age, educational status, relationship status and occupational status were the determinants for male partner involvement in PMTCT services [18, 19].

Despite this pivotal role played by male partners, evidences currently suggests that, their level of involvement is currently low in sub Saharan African [20, 21]. There is widespread use of Antiretroviral Therapy (ART) by pregnant women living with HIV but the rate of MTCT of HIV in low-middle income countries including Ethiopia is still high. In Ethiopia, 3.8–18% of the children born with HIV infected mothers were positive for HIV [21–24].

## Methods and materials

### Reporting and protocol registration

This systematic review and meta-analysis was reported based on the guideline of reporting systematic review and meta-analysis (PRISMA).

### Searching strategy

We have used PubMed, Google scholar and free Google databases search engines. In addition, we have used search strings adapted to the requirements of each database. We had used the key words ((“male partner involvement” [MeSH Terms] OR “partner involvement” [All Fields]), “associated factor” [MeSH Terms] OR: determinants” [All Fields])) and (“Attending PMTCT service”) to search under PubMed/Medline search engine.

### Inclusion criteria

Studies conducted since 2013 among male partners of HIV positive mothers were included in the study.

### Exclusion criteria

Those articles which have no full information (no reporting of either magnitude or result of multiple logistic regression analysis of male partner involvement in PMTCT service) were excluded from the study.

### Extraction of data from eligible papers

Data were extracted using the standardized data extraction tool in considering the name of the first author, date of publication, study setting, target population, study region, study area, study design, sample size, status of male partner involvement and determinants [risk estimate (OR) and their 95% confidence interval]. The data extraction was done independently by two reviews. Disagreements were resolved by revising, discussing and finally reaching to a common consensus.

### Quality assessment for studies

Study quality was assessed using a standardized tool adapted from the NEWCASTLE–OTTAWA QUALITY ASSESSMENT SCALE for crossectional studies which is adapted from Newcastle–Ottawa Quality Assessment Scale for cohort study. The tool considered the following study characteristics: sampling representative and size, non-respondents, and ascertainment of the exposure (risk factor). Studies fulfilling the required criteria as score 1 and studies with scores 0 were considered to be poor quality for specified criteria. No study was excluded from the review based on their quality scores.

### Data management and processing

The outputs from the searching engines were imported into Endnote Version × 6 software and duplicates were removed. Data were recorded on the abstraction forms and entered into Revman 5.4 for analysis.

### Data synthesis and analysis

Both systematic review and meta-analysis were done by using Revman 5.4 software. In the qualitative part of the review, all eligible articles reporting as male partner involvement among partners of HIV positive mothers attending PMTCT service and their determinant factors were summarized. Meta-analyses (quantitative reviews) were conducted to determine the overall pooled magnitude of male partner involvement during Prevention of Mother to Child Transmission (PMTCT) service delivery. Heterogeneity was evaluated using the Cochran statistic and the I2 statistics. The magnitude of statistical heterogeneity between studies was assessed using I^2^ statistics and values of 25, 50 and 75% were considered to represent low, medium, and high, respectively. The random-effects model was used for the data identified as heterogeneous during analysis. Meta-analyses and meta-regression were performed using Revman. For the magnitude of male partner involvement with data from 11 studies, we performed meta-regression analyses to calculate the odd ratio (OR).presence of publication bias was assessed by a funnel plot.

## Results

### Search results

**Fig. 1:**
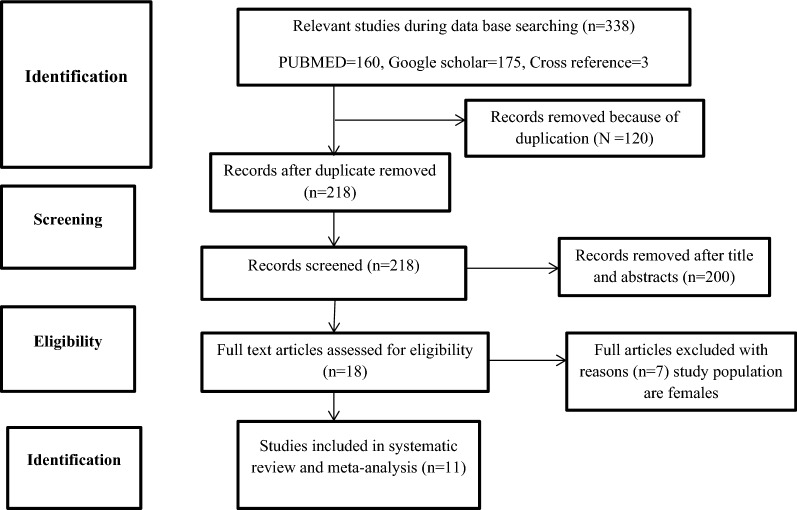
PRISMA flow diagram for the studies screened, reviewed, and included in Ethiopia, 2021

The combined literature search strategy retrieved a total of 338 potential studies, of which 3 records were articles by manual search sources, 18 were screened for full-text review and 11 studies were eligible to be included in the systematic and meta-analysis (Fig. 1).

### Description and risk bias assessment of the included studies

Eleven articles that were published in different parts of Ethiopia from international peer reviewed and national journals included estimating magnitude and determinants of involvement of male partner during PMTCT activities. The total sample size from these 11 included studies was 5129. Among a total of study participants, 2040 of them were involved in PMTCT activities. The sample size of included studies ranged from 210 which is the minimum sample size [8] to 802 which is the maximum sample size [2]. All of the reviewed studies were crossectional studies. The highest and lowest magnitude of male partner involvement among study regions were in Addis Abeba [8] and Amhara regions [25], which was 10% and 72% respectively (Table 1).

**Table 1:** Description of included articles for the study determinants of male involvement in PMTCT service utilization in Ethiopia (2013–2021)

The author with a publication year	Study design	Study region	Study population	Sample size	P in (%)	Study quality
Adane et al. 2020 [26]	crossect	Amhara	Male partner	525	26.1	Good
Bedru et al. 2019 [8]	crossect	A.A	Male partner	210	10	Good
Degefa et al. 2017 [27]	crossect	SNNP	Male partner	401	30.9	Good
Endawek et al. 2013 [25]	crossect	Amhara	Male partner	274	72.3	Good
Eriste et al. 2020 [28]	crossect	Oromia	Male partner	374	42.5	Good
Haile et al. 2021 [29]	crossect	SNNP	Male partner	605	53.7	Good
Maregn et al. 2015 [10]	crossect	SNNP	Male partner	720	53.0	Good
Tekle et al. 2021 [30]	crossect	SNNP	Male partner	402	52.4	Good
Worku et al. 2018 [31]	crossect	Oromo	Male partner	405	52.1	Good
Zeytuna et al. 2021 [32]	Crossect	A.A	Male partner	411	25.1	Good
Abdulfeta et al. 2016 [2]	crossect	AA	Male partner	802	20.9	Good

### Magnitude of male partner involvement

**Fig. 2:**
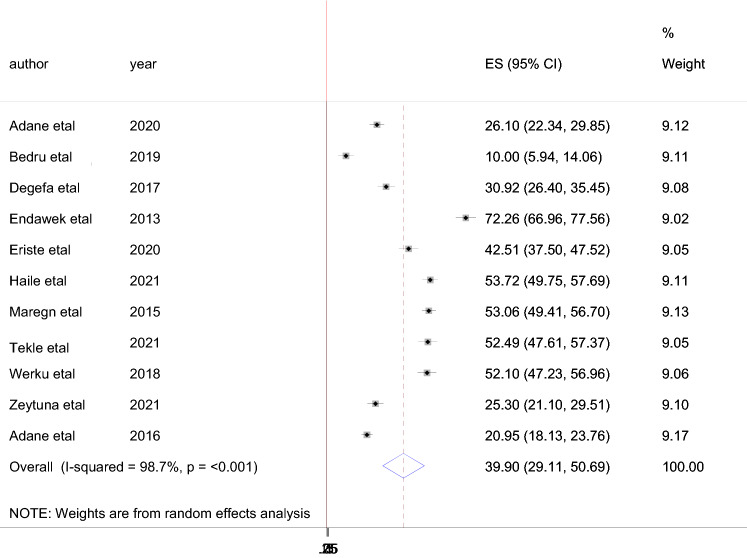
Magnitude of male partner involvement in PMTCT activities in Ethiopia (2016–2021)

Forest plot was plotted to estimate the pooled prevalence of male partner involvement. The pooled prevalence of male partner involvement among male partners of HIV positive mothers attending PMTCT service was 40% (95% CI: 29.11–50.69) with I^2^ = 98.7%, p ≤ 0.001 (Fig. 2).

### Publication bias

**Fig. 3:**
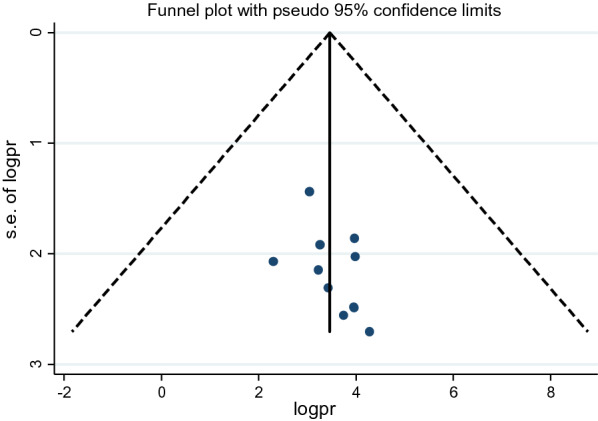
Funnel plot test for study of determinants of male involvement in PMTCT service utilization in Ethiopia (2013–2021)

The funnel plot test showed that there is no evidence of substantial publication bias for magnitude of male partner involvement in Ethiopia (Fig. 3).

### Factors associated with male partner involvement

Forest plot was run to assess factors which were significantly associated with male partner involvement. The forest plot showed that knowledge of husband on PMTCT (2.30, 95% CI 1.75, 3.02), perceived responsibility of women only (4.22, 95% CI 2.31, 7.71), being government employee (2.89, 95% CI 2.02, 4.12), cultural barriers, (3.44, 95% CI 2.54, 4.65) and educational status of husband (2.4, 95% CI 1.79, 3.50) were determinants of male partner involvement (Table 2).

**Table 2:** A meta-analyses of determinants of male partner involvement among in mothers attending PMTCT service in Ethiopia (2013–2021)

Variables	OR (95% CI)	I^2^	Tau^2^	P Value of Tau^2^	Q statistics
Knowledge on PMTCT					
Good	3.70 (2.80–4.89)	31	0.03	0.23	4.35
Poor (Reff)	1				
Perceiving the responsibility is for female only					
Yes (Reff)	1				
No	4.22 (2.31–7.71)	0.04	75	0.14	4.02
Educational status					
Grade 1–8 (Reff)	1				
> 8	2.51 (1.79–3.5)	66	0.08	0.03	8.95
Government employee					
Yes	2.89 (2.02–4.12)	< 0.001	< 0.001	< 0.001	< 0.001
No (Reff)	1				
Cultural barriers					
High (Reff)	1				
Low	3.44 (2.54–4.65)	< 0.001	< 0.001	0.88	0.02

Reff = reference

The odds of male partner involvement who had knowledge on PMTCT was 2 times more likely to be involved in PMTCT activities as compared with the counter parts (OR: 2, 95% CI 1.75, 3.02). Male partners who were government employee were 3 folds higher to be involved in PMTCT of HIV/AIDS than those who were not government employee (OR 2.89, 95% CI 2.02, 4.12). The odd of being involved in PMTCT services among male partners who were attended grade 9 and above were 2.5 times higher as compared with those who were attended grade 8 or below (OR 2.4, 95% CI 1.79, 3.50) (Table 2).

## Discussion

This study was assessed the pooled magnitude of male partner involvement and its determinant among male partners of mothers who are attending PMTCT service in Ethiopia. From the total study participants, only (40%) of them were involved in PMTCT Activities. This is lower than the level in sub Saharan Africa which is 48% [9]. This might be due to the difference in economic, social and health service delivery condition varies from country to country even from region to region.

Knowledge of male partner on PMTCT became the determinant for involving in PMTCT activities. This study finding was consistent with study conducted in Tanzania [34]. This might be due to having knowledge on PMTCT will enable the partners to know about the consequence of non-utilizing of PMTCT service on his wife and on his new born infant.

Educational status is also significantly associated with male partner involvement. Those male partners who attend grade nine and above were more likely to be involved in PMTCT of HIV/AIDS than male partner who attend grade eight or less. Educated individuals are more knowledgeable on consequence of HIV/AIDS than their counter parts. This is in-line with the fact that people that are more knowledgeable could take care of HIV infection, as they easily understood both the transmission and prevention methods. Similarly, other studies conducted in Uganda and elsewhere have indicated that education level is an important determinant of participation in PMTCT services [15, 35].

This study showed that male partner who perceive as “attending PMTCT service is the role of women only” were less likely to participate in PMTCT service. This might be because of that if male partner attitude is not positive for PMTCT service, they will be not volunteer in supporting financial (for transport), sharing work load and, psychological support.

In this study government employees were more likely to involve in PMTCT activities than being in non-government employee. This finding is similar with study finding of Addis Ababa [36] and Uganda [35]. The possible explanation for this might be that those government employers were more educated and had more awareness about health related issues than private employers like daily laborer [2].

This study showed that presence of cultural barrier is determinant for male partner involvement in PMTCT service which is supported by studies conducted in Ethiopia, Uganda and Tanzania [2, 35, 37]. The possible explanation might be those males who had low cultural barriers can accompany their partners during all maternal and child health services, and communicate with their wives freely about the service obtained from the PMTCT program. Social and religious norm could prohibited males from attending female health services, and the widespread attitude that female reproductive health is not male responsibility found to inhibit male involvement in PMTCT service [38].

## Conclusion

The current study showed that the pooled prevalence of male partner involvement was lower than study conducted in sub Saharan Africa. Knowledge of husband on PMTCT, perceiving “the responsibility is for women only”, being government employee, cultural barriers, and educational status of husband were determinants of male partner involvement. Therefore, the existing strategies to improve male involvement should be strengthened.

## Data Availability

The data is available at reasonable request.
